# Test characteristics of two rapid antigen detection tests (SD FK50 and SD FK60) for the diagnosis of malaria in returned travellers

**DOI:** 10.1186/1475-2875-8-90

**Published:** 2009-05-05

**Authors:** Mirna Van der Palen, Philippe Gillet, Emmanuel Bottieau, Lieselotte Cnops, Marjan Van Esbroeck, Jan Jacobs

**Affiliations:** 1 Faculty of Health, Medicine and Life Sciences (FHML), Maastricht, The Netherlands; 2 Department of Clinical Medicine, Institute of Tropical Medicine (ITM), Antwerp, Belgium

## Abstract

**Background:**

Two malaria rapid diagnostic tests were evaluated in a travel clinic setting: the SD FK50 Malaria Ag *Plasmodium falciparum *test (a two-band test) and the SD FK60 Malaria Ag *P. falciparum*/Pan test (a three-band test).

**Methods:**

A panel of stored whole blood samples (n = 452 and n = 614 for FK50 and FK60, respectively) from returned travellers was used. The reference method was microscopy with PCR in case of discordant results.

**Results:**

For both tests, overall sensitivity for the detection of *P. falciparum *was 93.5%, reaching 97.6% and 100% at parasite densities above 100 and 1,000/μl respectively. Overall sensitivities for *Plasmodium vivax, Plasmodium ovale *and *Plasmodium malariae *for the FK60 test were 87.5%, 76.3% and 45.2%, but they reached 92.6% and 90.5% for *P. vivax *and *P. ovale *at parasite densities above 500/μl. Specificities were above 95% for all species and both tests when corrected by PCR, with visible histidine-rich protein-2 lines for *P. malariae *(n = 3) and *P. vivax *and *P. ovale *(1 sample each). Line intensities were reproducible and correlated to parasite densities. The FK60 tests provided clues to estimate parasite densities for *P. falciparum *below or above 1,000/μl.

**Conclusion:**

Both the FK50 and FK60 performed well for the diagnosis of *P. falciparum *in the present setting, and the FK60 for the diagnosis of *P. vivax *and *P. ovale *at parasite densities > 500/μl. The potential use of the FK60 as a semi-quantitative estimation of parasite density needs to be further explored.

## Background

Microscopic diagnosis of malaria requires considerable training and experience. Most diagnostic laboratories in non-endemic countries lack sufficient samples to enable building-up and maintenance of microscopic expertise. In addition, many returned travellers suspected of malaria present outside office hours, when expert microscopy may not be at hand [1,2]. Rapid diagnostic tests (RDTs) offer a simple and rapid complement to microscopic malaria diagnosis. The earlier two-band tests were designed to detect *Plasmodium falciparum*. They display a control line and a test line, which targets either histidine-rich protein-2 (HRP-2) or *P. falciparum*-specific parasite lactate dehydrogenase (pLDH). Newer generation three-band tests display a control line and two test lines, one for detection of *P. falciparum*-specific antigen and another for detection of antigens common to the four species, such as pan-*Plasmodium*-specific parasite lactate dehydrogenase or aldolase. Simplified "one-step" malaria RDTs have been marketed. Unlike their predecessors, the one-step RDTs only require one or two manipulations, *i.e*. application of blood and a running buffer. It is to be expected that they will increase performance by laboratory and clinical staff who are using the RDTs on an incidental base. Indeed, multistep RDTs have been demonstrated to require considerable training to reach optimal sensitivity [2]. Many brands are marketed, but published reports are only available for a small number of them [3-6]. The World Health Organization, through the Regional Office for the Western Pacific, lists a number of malaria RDTs, which are produced in compliance with ISO 13485:2003 [7].

The SD FK50 Malaria Ag *P. falciparum *test (Standard Diagnostics, Hagal-Dong, Korea) and the SD FK60 Malaria Ag *P. falciparum*/Pan (Standard Diagnostics) are one-step malaria diagnostic tests in a cassette format, in a two- and three-band design, respectively. In this study, their performance was assessed when challenged with a collection of stored blood samples of returned travellers in a reference centre. For convenience, these tests will be referred to as FK50 and FK60, respectively.

## Methods

### Study design

Both kits were retrospectively evaluated in a reference laboratory on a panel of stored blood samples obtained in returned travellers suspected of malaria. The reference method was microscopy, performed at presentation of the patient. All discordant results were subsequently analysed by PCR, and test characteristics were recalculated according to the PCR-corrected results.

### Patients and Materials

EDTA-blood samples from patients attending the outpatient clinic of the Institute of Tropical Medicine (ITM), Antwerp, Belgium, or those that were sent by Belgian laboratories to ITM for confirmation in the scope of the national reference function were used. Patients included European travellers returning from malaria-endemic areas and, to a lesser extent, natives of endemic countries returning from visiting friends and relatives. The samples were submitted as part of the diagnostic protocol for suspected malaria. Samples had been collected from January 1996 to October 2007 and had been stored at -70°C. Diagnosis was based on standard microscopy. Among these samples, a panel was selected based on relevant representation of the four malaria species (*P. falciparum*, *Plasmodium vivax*, *Plasmodium ovale *and *Plasmodium malariae*) and different parasite densities. For the FK60, additional samples of non-falciparum species were included. Samples without malaria parasites (negative samples) were collected prospectively during the period of September and October 2007 from returned travellers attending the outpatient clinic of ITM and for whom a thick film, requested as part of work-up of suspected malaria, did not show any malaria parasites.

### Reference method

Diagnosis of malaria, species identification and determination of parasite density were done by microscopy. According to standard practice at the ITM, thick and thin blood films were prepared, stained with Giemsa (pH 8.0) and examined by light microscopy using a × 500 magnification. An examination of 15 minutes for a thick film, with a minimum of 200 fields read, was performed before the blood film was reported negative. Parasite densities were estimated by counting asexual parasites against 200 white blood cells (WBC) in thick blood films, converting this number to parasites/μl using the actual WBC count or, when this was not available, the standard 8,000 WBC/μl value [4]. Parasite densities are further in this text expressed as counts (of asexual parasites)/μl (of whole blood).

### Test platforms

The FK50 is a two-band RDT targeting HRP-2 antigen. Results are expressed as positive or negative for *P. falciparum*. The FK60 is a three-band test targeting HRP-2 and pLDH. The presence of a HRP-2 line together with a pLDH line indicates an infection with *P. falciparum *or a mixed infection with *P. falciparum *and one or more of the other *Plasmodium *species. The presence of a unique HRP-2 line refers to an infection with *P. falciparum*, whereas a unique pLDH line indicates infection with one or more of the other *Plasmodium *species. Both assays are lateral flow immunochromatographic antigen detection tests in a cassette format.

### Test procedure

Tests were performed according to the instructions of the manufacturer, except that samples (5 μl) were loaded with a transfer pipette (Finnpipette, Helsinki, Finland) instead of the plastic tube supplied by the manufacturer and that a scoring system was used to assess the intensity of the test lines. In cases for which the control line did not appear, the results were interpreted as invalid and the tests were repeated. In order to score test line intensities, the scoring system of Bell and co-workers [8]was used and five categories were defined: None (no line visible), Faint (barely visible line), Weak (paler than the control line), Medium (equal to the control line) or Strong (stronger than the control line). To assure timely readings, tests were carried out in time-controlled batches of five samples. Readings were performed by three subsequent observers, of whom the one who performed the test procedure invariably was the first. Observers were blinded to the results of microscopy and to each others' readings. Readings were carried out at daylight assisted by a standard electricity bulb, between 20 and 30 minutes after application of the sample and buffer. The results of the readings considered were based on consensus agreement, which means that the same result was observed by at least two out of three different observers. Where there was no consensus (in rare cases for the FK60), results of the first observer were considered. To assess inter-observer agreement, results of positive and negative readings as well as line intensity readings were considered. To assess test reproducibility, a panel of six positive samples for *P. falciparum *with various parasite densities (116, 200, 1,123, 2,900, 138,000 and 275,000/μl) was tested on five occasions.

### Statistical analysis

For the FK50, true positive results were defined as those with a HRP-2 line visible in samples with *P. falciparum *seen at microscopy, and true negative results as those with no HRP-2 line visible in microscopy-negative samples. Incorrect test results included false-negative samples (those with a microscopic diagnosis of *P. falciparum *but no test line visible), false-positive samples (microscopic negative samples showing a HRP-2 line) and species misidenfications (non-falciparum species showing a HRP-2 line). For the FK60, samples infected with *P. falciparum *and the non-falciparum species were considered separately and the control panels included both microscopy negative samples and samples infected by the non-falciparum species and *P. falciparum *respectively, as shown in Tables 1 and 2. Of note is that, for both tests, samples with pure gametocytaemia were included among the positive *P. falciparum *samples. To avoid complex problems of interpretation, microscopically identified mixed infections were considered separately and not included in the calculations of test characteristics.

**Table 1:** Interpretation of test results of the FK60 for the detection of *P. falciparum *infection

	Microscopy result	
	
Test line(s) visible	*P. falciparum* (asexual and/or gametocytes)	*P. vivax*, *P. ovale *or *P. malariae *or no parasites seen
		
		
		Incorrect results:
Unique HRP-2 line or Both HRP-2 + pLDH lines	True-positives	- False positive reactions among "no parasites seen" - Species misidentifications (non-falciparum species diagnosed as *P. falciparum*)
	Incorrect results:	
No test line or Unique pLDH line	- False negative reactions among *P. falciparum *samples - Species misidentification (*P. falciparum *diagnosed as non-falciparum species)	True-negatives

**Table 2:** Interpretation of test results of the FK60 for the detection of *Plasmodium *non-falciparum infection

	Microscopy result	
	
Test line(s) visible	*P. vivax, P. ovale *or *P. malariae*	*P. falciparum *or no parasites seen
		
		Incorrect results:
Unique pLDH line	True-positives	- False positive reactions among "no parasites seen" - Species misidentifications (*P. falciparum *diagnosed as non-falciparum species)
		
	Incorrect results:	
No test line or Unique HRP-2 line or Both pLDH + HRP-2 lines	- False negative reactions among non-falciparum samples - Species misidentification (non-falciparum species diagnosed as *P. falciparum*)	True-negatives

Sensitivity and specificity were calculated with 95% confidence intervals (C.I.) and differences were tested for significance using the chi-square test or, in case of small sample sizes, a two-tailed Fisher's exact test. A p value < 0.05 was considered as significant. In addition, positive and negative likelihood ratios (LHR+ and LHR-) were calculated. Likelihood ratios provide direct information on the tests power to include (LHR+ > 10) or exclude (LHR- < 0.1) a disease without being influenced by its prevalence [3]. Inter-observer agreements were assessed using the kappa statistic for paired observers and percentage agreements for all three observers combined. Associations between line intensity readings and parasite densities were assessed for strength of association with Cramer's *V *for categorical variables, using interpretative criteria published previously [9].

### Analysis of discordant results

For discordant results (*i.e*. false-negative and false-positive results and species misidentifications) a species-specific polymerase chain reaction (PCR) was performed. Primers and probes sequences detecting small subunit 18S rRNA genes were selected according to Rougemont *et al *[10].

### Ethical review

The study was reviewed and approved by the Institutional Review Board of ITM and by the Ethical Committee of Antwerp University, Belgium.

## Results

### SD FK50 Malaria Ag *P. falciparum *test

#### Sample collection

A total of 452 samples were assessed, including those infected with *P. falciparum *(n = 324), *P. vivax *(n = 12), *P. ovale *(n = 11), *P. malariae *(n = 6), mixed species (n = 4) and negative samples (n = 95). The majority (272/324, 84.0%) of *P. falciparum *samples had been acquired in sub-Saharan Africa.

#### Sensitivity, specificity and likelihood ratios

No invalid test results were observed. Additional file 1 shows the overall and detailed test characteristics matched for parasite densities. Sensitivity increased marginally when samples with pure gametocytaemia were subtracted. Sensitivity was related to parasite density, with values at parasite densities < 100/μl significantly lower compared to those at parasite densities > 100/μl (78.9% and respectively 97.6%, p < 0.001). Fifteen out of 21 false-negative samples had parasite densities < 100/μl (including three samples with pure gametocytaemia), the remaining six had parasite densities ranging from 122 to 400/μl. All these infections had been acquired in Africa. Of the four samples with mixed infection, a single one (with *P. falciparum *and *P. ovale*) gave a false-negative result. The overall specificity was 94.4%, with seven samples that were incorrectly identified: a visible test line was observed among four microscopic negative samples and three *P. malariae *samples. The exclusion power was excellent (LHR- < 0.10) except for parasite densities less than 100/μl, the inclusion power was also excellent (LHR+ > 10).

#### Line intensity readings

Table 3 lists the line intensity readings related to parasite density. Line intensity readings were significantly related to parasite densities with a substantial correlation (*V *= 0.434, p < 0.001), but there was considerable overlap between categories. Faint or weak line intensities occurred in 98/305 (32.1%) true positive results, mostly but not exclusively at low parasite densities and among all seven false positive results and misidentifications.

**Table 3:** Line intensity consensus readings for the FK50 according to parasite densities

	Line intensity readings, numbers of samples*					
		
**Result by microscopy**	Negative	Faint	Weak	Medium	Strong	**Total**
						
*P. falciparum*						
All samples combined, total	22	4	94	66	141	327
Samples with only gametocytes	3	1	8	4	1	17
Asexual parasite density 0–100/μl	12	2	32	9	2	57
Asexual parasite density 101–200/μl	3		21	8	3	35
Asexual parasite density 201–1000/μl	4	1	28	28	20	81
Asexual parasite density > 1000/μl			5	17	115	137
						
Other species and no parasites seen, total	118	4	3			125
No parasites seen	91	3	1			95
*P. vivax*	13					13
*P. ovale*	11					11
*P. malariae*	3	1	2**			6

* Total number of samples = 452, including two *P. falciparum/P. ovale *and one *P. falciparum/P. malariae *mixed infections (allocated under *P. falciparum*) and one *P. vivax/P. malariae *mixed infection (allocated under *P. vivax*)

**PCR confirmed microscopic diagnosis for all discordant samples except for one sample that was diagnosed as a mixed *P. malariae*/*P. falciparum *infection

#### Inter-observer agreement and reproducibility

The inter-observer agreement for positive and negative readings was high, with 97.3% overall agreement between the three observers and kappa values between 0.95–0.98 for each pair out of three observers. All discordances in line intensity readings were limited to one category of difference (*e.g*. a line scored as weak by one observer was scored as medium or faint but not as strong by the other observer(s)). For line intensity readings, overall agreement was 86.9% and kappa values were between 0.86 and 0.90. Reproducibility testing showed consistent readings by all three observers on all occasions for three out of six samples. For two other samples discrepancies were limited to differences within one category of line intensity and had no impact on final test result. A single sample (with parasite density 1,123/μl) provided weak, faint and negative readings upon repeat testing.

#### Analysis of discordant results

PCR analysis confirmed the discrepant results in favour of the reference microscopy except for a single sample that was microscopically identified as *P. malariae *and while showing a HRP-2 line, this sample was diagnosed by PCR as a mixed *P. falciparum/P. malariae *infection (Additional file 1). Correcting for this result increased the specificity from 94.4% to 95.2% (C.I. 89.6%–98.0%).

### SD FK60 Malaria Ag *P. falciparum*/Pan test

#### Sample collection

Compared to the panel for the FK50, the numbers of samples with non-falciparum species were increased up to 80 for *P. vivax *and *P. ovale *each and 31 for *P. malariae*, resulting in a total of 614 samples.

#### Sensitivity, specificity and likelihood ratios

No invalid test results were recorded. A total of 303 out of 324 *P. falciparum *samples were correctly identified. The remaining 21 samples showed no test line. Among the control samples, there was one out of 95 microscopy negative samples that generated a HRP-2 line, and there were six out of 191 non-falciparum samples showing cross-reaction with HRP-2 (one sample each with *P. ovale *and *P. vivax *and four samples with *P. malariae*). The resulting test characteristics are displayed in Additional file 2, with an overall sensitivity and a specificity of 94.1% and 97.6% respectively. As for the FK50, sensitivities were lower at decreasing parasite densities with significant difference below and above 100/μl (p < 0.001).

For the *P*. non-falciparum species, there were 145 out of 191 samples correctly identified (unique pLDH line visible), six samples had a visible HRP-2 line and 40 samples showed no test line. The resulting overall sensitivities were 87.5%, 76.3% and 45.2% for *P. vivax*, *P. ovale *and *P. malariae *respectively (Additional file 3). Sensitivity for *P. malariae *was significantly lower compared to the two other species (p < 0.001). As for *P. falciparum*, sensitivities declined at lower parasite densities, and dipped at parasite densities below 500/μl. Differences between sensitivities in samples above and below parasite densities of 500/μl reached statistical significance in the case of *P. ovale *(p < 0.05). Among the microscopic negative samples, there were no false-positive pLDH readings neither were there *P. falciparum *samples that generated a unique pLDH line. The specificity for non-falciparum species was 100%, resulting in high LHR+ values.

Of the four samples with mixed infection, a single one (with *P. falciparum *and *P. ovale *at a parasite density of 700/μl) showed only a pLDH line, the other mixed infections were correctly identified.

#### Line intensity readings

As for the FK50, faint and weak line intensities for the HRP-2 line occurred mostly but not exclusively at low parasite densities. The pLDH line performed worse in terms of visibility, with 224/353 (63.4%) of true positive readings in the faint or weak categories, as opposed to 90/305 (29.5%) for the HRP-2 line (p < 0.001). The pLDH line showed lowest intensities among the non-falciparum samples, with only 28 out of 80 *P. vivax *samples, 5 of 80 *P. ovale *samples and none of the *P. malariae *samples showing medium or strong line intensities. Line intensity readings for HRP-2 and for pLDH were related to parasite densities (HRP-2: *V *= 0.387, p < 0.05; pLDH: *V *= 0.457, p < 0.05) but there was considerable overlap between the different categories. Interestingly, in the case of *P. falciparum *infection, the unique presence of a HRP-2 line pointed almost exclusively (98.1%, 103/105 of samples) to a parasite density below 1,000/μl (Table 4). In addition, the presence of medium or strong pLDH line intensity was invariably associated with parasite densities exceeding 1,000/μl in the case of *P. falciparum *and 500/μl in the case of the non-falciparum species, except for one *P. falciparum *sample with pure gametocytaemia (12,700/μl). Of interest, among the 17 *P. falciparum *samples with pure gametocytaemia, there were 14 with HRP-2 lines visible compared to six with pLDH lines visible. HRP-2 lines in samples with non-falciparum species gave faint or weak line intensities except for a single *P. malariae *sample with strong line intensity. This latter sample however proved to be a mixed infection with *P. falciparum*/*P. malariae *upon PCR analysis (see below).

**Table 4:** Reactivity of test lines for the FK60 for *P. falciparum *samples in relation to parasite density

	SD FK 60 result: test lines visible			Total
		
Parasite densities for *P. falciparum *samples	None	Unique HRP-2 line	Both HRP-2 and pLDH lines	
				
Pure gametocytaemia	3	8	6	17
0 – 100/μl	12	42	3	57
101 – 200/μl	3	18	14	36
201 – 1000/μl	3	35	42	80
> 1000/μl	0	2	133	135
Total	21	105	198	324

#### Inter-observer agreement and reproducibility

Both target lines performed well for inter-observer agreements, although the results of the HRP-2 line were better than the pLDH line (Table 5). Most discordances in line intensity readings occurred within one category of difference (81/85 (95.3%) and 125/129 (96.9%) for HRP-2 and pLDH respectively). The results for the reproducibility testing for the HRP-2 line were comparable to those obtained in the FK50 test. For the pLDH line, consistent readings were recorded by all three observers on all occasions for four out of six samples. Two other samples (with parasite density 116/μl and 200/μl) provided weak, faint and negative readings upon repeat testing.

**Table 5:** Overall agreement and inter-observer agreement between pairs of observers for the FK60 assay

	Overall agreement between three observers (%)	Agreement between pairs of observers (kappa values)
		
Results expressed as positive and negative readings		
		
HRP-2	96.9	0.96, 0.95, 0.96
pLDH	91.9	0.88, 0.88, 0.91
		
Results expressed as line intensity readings		
		
HRP-2	86.2	0.86, 0.86, 0.87
pLDH	79.0	0.79, 0.80, 0.80

#### Analysis of discordant results

PCR analysis of all discordances resulted in the following corrections: two samples that were microscopically diagnosed as *P. ovale *and that did not show any test line proved to be mixed *P. falciparum/P. ovale *infections, and one *P. malariae *sample that showed cross-reaction with the HRP-2 line proved to be a mixed *P. falciparum/P. malariae *infection. When correcting for these results, specificity for the detection of *P. falciparum *and sensitivity for the diagnosis of *P. malariae *increased slightly (Additional files 2 and 3).

### Side by side comparison of the SD FK50 and SD FK60 assays

A total of 324 *P. falciparum *samples were assessed by both the FK50 and FK60 tests. Two of them provided different results: one was uniquely positive by FK50 and the other by FK60. Both samples had parasite densities below 100/μl, and displayed faint and weak HRP-2 line intensity readings without a visible pLDH line. Since both samples belonged to the same parasite density category (< 100/μl), data on sensitivity for *P. falciparum *were identical for both assays. In addition to the three *P. malariae *samples that showed HRP-2 lines with the FK50 test, the FK60 showed HRP-2 lines for three other samples, one sample of *P. malariae, P. vivax *and *P. ovale *respectively. Among microscopic negative samples, a single sample gave a false-positive HRP-2 line by the FK60 as opposed to four samples for the FK50, resulting in slightly better specificity and LHR+ for the FK60 as compared to the FK50. Agreement between both tests for positive and negative readings and line intensities was excellent (kappa value 0.95) and substantial (kappa value 0.68) respectively.

## Discussion

In this study, the performance of two one-step malaria rapid diagnostic tests, the SD FK50 Malaria Ag *P. falciparum *test (a two-band HRP-2 test) and the SD FK60 Malaria Ag *P. falciparum*/Pan test (a three-band HRP-2 and pLDH test) was evaluated on large panels of stored samples obtained from international travellers. For both tests, overall sensitivity for the detection of *P. falciparum *was 93.5%, reaching 97.6% and 100% at parasite densities above 100 and 1,000/μl respectively. Overall sensitivities for *P. vivax, P. ovale *and *P. malariae *for the FK60 test were 87.5%, 76.3% and 45.2%, but they reached 92.6% and 90.5% for *P. vivax *and *P. ovale *at parasite densities > 500/μl. Specificities were above 95% for all species. Inter-observer agreement and reproducibility were high for both tests.

One of the limitations of the present study was its retrospective design, which made it difficult to trace back causes of discordant results such as previous therapy and the presence of interfering factors (*e.g*. rheumatoid factor). Further, the study population was not completely homogenous and the small numbers of semi-immune immigrants (who may tolerate low-level parasite densities [5]) were not identified. Another possible limitation is the fact that stored blood samples were used for analysis. Although, on theoretical grounds, there have been concerns about the stability of the target antigens under these conditions [11], previous evaluations of RDTs have been performed on stored samples [12,13] and a prospective evaluation of fresh and stored samples revealed similar results in case of the HRP-2 antigen [14]. In the present study, no obvious differences in test performance were found for samples stored for several (> 2) years compared to those stored for a shorter period (results not shown), and the samples had not been exposed to repeat thawing and freezing. Further, it should be realised that the present study design did not consider the performance of these RDTs when applied in clinical diagnosis by laboratory technicians in non-endemic settings, who have few exposure to malaria positive samples [1,2]. Assessing samples with different parasite densities should be part of the laboratory validation when introducing RDTs in clinical practice, for instance, to train the occasional reader to interpret faint line intensities as positive results. Finally, it should be realized that the present study was performed in a reference setting, with the availability of expert microscopy, trained observers and optimal environmental conditions. Likewise, a calibrated transfer pipette was used instead of the manufacturer's transfer device, in order to ensure correct volume transfer [11]. However, an evaluation of such a test in a reference setting is a logic first step preceding in-depth evaluations and field trials [11].

For both tests and the diagnosis of *P. falciparum*, the sensitivities were in line with those reported in other HRP-2 tests in returned travellers, with sensitivities ranging from 80% to 99%, depending on the setting and parasite densities [3,15-22]. However, most of these studies, in particular the systematic reviews, addressed the multistep RDTs that are available on the market for a long time [3,5,6,23]. By contrast, evaluations of most of the other RDTs displayed on the WHO website are pending [7]. For the non-falciparum species, the reported sensitivities vary, with decreasing sensitivities for *P. vivax *followed by either *P. ovale *or *P. malariae *[3,4,15,19,24]. In a recent meta-analysis on malaria RDTs in international travellers [3], sensitivities for *P. ovale *and *P. malariae *ranging from 36 – 95% were mentioned. Part of this wide range in sensitivities is probably due to low sample sizes in different studies. The sample sizes in the present study enabled us to calculate test characteristics with narrower confidence intervals, and consequently this study demonstrates a significantly lower sensitivity for detection of *P. malariae*, as compared to *P. ovale *and *P. vivax*, even at parasite densities above 500/μl. Considering the present methods, two other remarks are to be made. First, in the case of *P. falciparum*, samples with only gametocytes were considered as part of the microscopic positive samples. From the standpoint of travel medicine, this is a recommended choice, but gametocytes may be present even after successful eradication of the asexual forms [3,5]. Moving these pure gametocytaemia samples to the "non-malaria" category in the present study collection would add slightly to the sensitivity and the LHR- of both tests (Additional files 1 and 2), at a considerable cost of specificity (87.0% and 93.3% for FK50 and FK60 respectively), but with LHR+ still above 10. Second, among our control panel not only microscopy negative samples were included, but also samples from other *Plasmodium *species and we consequently scored species misidentifications as incorrect diagnosis. One could argue that species misidentification can be tolerated as long as the diagnosis of malaria is not missed. Competent malaria diagnosis however requires distinction between at least *P. falciparum *and the other species, as prognosis, therapy, follow-up and epidemiology are different. With regard to both tests, it is reassuring that among the present samples, *P. falciparum *was not erroneously misidentified as a non-falciparum species (with the exception of a single failure of HRP-2 reactivity in a mixed *P. falciparum/P. ovale *infection), and that misidentification only occurred in the other direction, *i.e*. non-falciparum species (especially *P. malariae*) were misidentified as *P. falciparum*. The impact of adding other species to the control group however was low in terms of test characteristics: limiting the control panel to exclusively the negative samples would result in a slight increase in specificity (95.8% and 98.9% for the FK50 and FK60 in case of *P. falciparum *respectively), a slight increase in sensitivity for *P. vivax *and *P. ovale *(88.8% and 77.5% respectively) and a moderate increase in sensitivity for *P. malariae *(58.1%). Of note are the false positive reactions for the HRP-2 line among the non-falciparum species, in particular among 10% of our *P. malariae *samples. HRP-2 cross-reaction have been reported for *P. vivax *and *P. malariae*, but not for *P. ovale *[25,26].

For all species, declining sensitivities at lower parasite densities were observed. For *P. falciparum *this is a well-known phenomenon [3-6,11]. The present study demonstrated this decline for the non-falciparum species as well, indicating a breakpoint at 500/μl. In line with the results from the meta-analysis mentioned above [3], most of the *P. falciparum *false-negative results in this study occurred in samples with parasite densities < 100/μl. Although the failure to detect high parasite densities, is also mentioned as a pitfall of malaria RDTs [3-6], no cases of false-negative results were presently found at parasite densities above 400/μl. It should be noted however that false-negative results at elevated parasite densities are rare events that await prospective surveillance by incident reporting. Further, the results did not show any relation between geographic distribution and false-negative results by HRP-2 due to possible genetic variations in HRP-2 target [27], but the majority of samples were acquired in Africa, and more samples should be tested from the Asia-Pacific to rule out an influence of such variations.

In contrast to most other studies, reproducibility and inter-observer agreements of both tests were presently assessed. Line intensity readings (and consequently test results) showed high inter-observer agreements and were also reproducible upon repeat testing, but performances were clearly better for the HRP-2 line as compared to the pLDH line. For the latter line, the preponderance of weak and faint readings for the non-falciparum species is of concern. Furthermore, for the detection of *P. falciparum*, the three-band FK60 test performed as well as the two-band FK50. This is of note, as one could expect the three-band test, which has to meet two optimums of antigen-antibody interactions, would perform somewhat less than the two-band test. Although the present devices are not designed to use line intensities as a tool for grading parasite densities, this study also explored their possible diagnostic value. In line with other findings, line intensities were related to parasite density [17,26,28] but considerable overlaps precluded their use as a semi-quantitative estimation of parasite density. However, the FK60 test provided interesting clues to parasite densities below or above 1,000/μl for *P. falciparum *(the unique presence of a HRP-2 line and the presence of medium or strong pLDH line respectively). A similar approach has been described for a HRP-2 and aldolase three-band RDT, for which co-reactivity of both test lines pointed to parasite densities of = 40,000/μl [29]. Further product research might refine and expand these possibilities, thereby enlarging the scope of malaria RDTs application [6].

## Conclusion

Taking into account their sensitivity and specificity, inter-observer agreement and reproducibility, it is clear that the FK50 and the FK60 tests devices are a valuable adjunct to microscopy for the diagnosis of malaria in a non-endemic setting. They share the limitations of other malaria rapid diagnostic tests, in particular the limited exclusion power for *P. falciparum *malaria at low parasite densities and a lower sensitivity for the non-falciparum species, especially *P. malariae*. Possible test improvements – apart from the sensitivity – would be an increase in intensity of the pLDH line, and the exploration of the semi-quantitative estimation of the parasite densities.

## Abbreviations

SD FK50: SD FK50 Malaria Ag *P. falciparum *test (Standard Diagnostics): a two-band "one-step" malaria rapid diagnostic test for the detection of *P. falciparum*, targeting HRP-2 antigen; SD FK60: SD FK60 Malaria Ag *P. falciparum*/Pan test (Standard Diagnostics): a three-band "one-step" malaria rapid diagnostic test for the detection of *P. falciparum *and *P*. non-*falciparum *species, targeting HRP-2 and pLDH antigen; Ag: Antigen; *P*.: *Plasmodium; *FHML: Faculty of Health Medicine and Life Sciences, Maastricht, The Netherlands; ITM: Institute of Tropical Medicine, Antwerp, Belgium; PCR: Polymerase Chain Reaction; RDT(s): Rapid Diagnostic Test(s); HRP-2: Histidine Rich Protein-2; pLDH: Parasite lactate dehydrogenase; EDTA: Ethylene diamine tetra-acetic acid; WBC: White Blood Cells; C.I.: Confidence interval; LHR+: Positive likelihood ratio; LHR-: Negative likelihood ratio; rRNA: Ribosomal ribonucleic acid; ∞: Infinite;

## Competing interests

The authors declare that they have no competing interests.

## Authors' contributions

MvdP, PG and JJ designed the study protocol. MvE and EB organized prospective sample collection. MvdP and PG carried out the test evaluations, LC performed PCR analysis. MvdP, PG and JJ analyzed and interpreted the results and drafted the manuscript, PG performed statistical analysis. All authors contributed to the discussion of the results and the redaction of the manuscript, they all approved the final manuscript.

## Supplementary Material

Additional file 1 **Test characteristics of the FK50 related to parasite densities.** Click here for file

Additional file 2 **Test characteristics of the FK60 for *P. falciparum *samples according to parasite densities.** Click here for file

Additional file 3 **Test characteristics of the FK60 for non-falciparum species according to parasite densities.** Click here for file
