# Supplementary material for: Test characteristics of two rapid antigen detection tests (SD FK50 and SD FK60) for the diagnosis of malaria in returned travellers
Source: Malar J. 2009 May 5;8:90. doi: 10.1186/1475-2875-8-90 (PMC2688521; doi:10.1186/1475-2875-8-90)
Supplement: Additional file 2 — Test characteristics of the FK60 for P. falciparum samples according to parasite densities. [file 1475-2875-8-90-S2.doc]

**Additional file 2**

**Test characteristics of the FK60 for *P. falciparum*** samples according to parasite densities (n = 610, 4 mixed infections not included)

| Result by microscopy |  | Numbers |  | Positive  by FK60* |  | Sensitivity in %  (95% C.I.) |  | Specificity in %  (95% C.I.) |  | Positive  Likelihood Ratio |  | Negative  Likelihood Ratio |
| --- | --- | --- | --- | --- | --- | --- | --- | --- | --- | --- | --- | --- |
|  |  |  |  |  |  |  |  |  |  |  |  |  |
| **Test characteristics for *P. falciparum* samples** |  |  |  |  |  |  |  |  |  |  |  |  |
| All samples combined |  | 324 |  | 303 |  | 93.5 (90.2-95.8) |  |  |  | 38.21 |  | 0.07 |
| Samples with only gametocytes subtracted |  | 307 |  | 289 |  | 94.1 (90.8-96.5) |  |  |  | 38.46 |  | 0.06 |
| Asexual parasite density 0-100/µl |  | 57 |  | 45 |  | 78.9 (66.6-87.7) |  |  |  | 32.26 |  | 0.22 |
| Asexual parasite density 101-200/µl |  | 35 |  | 32 |  | 91.4 (76.8-97.8) |  |  |  | 37.36 |  | 0.09 |
| Asexual parasite density 201-1000/µl |  | 80 |  | 77 |  | 96.3 (89.1-99.2) |  |  |  | 39.32 |  | 0.04 |
| Asexual parasite density > 1000/µl |  | 135 |  | 135 |  | 100 (97.6-100.0) |  |  |  | 40.86 |  | <0.01 |
| Asexual parasite density > 100/µl |  | 250 |  | 244 |  | 97.6 (94.6-99.0) |  |  |  | 39.88 |  | 0.03 |
|  |  |  |  |  |  |  |  |  |  |  |  |  |
| Other species and no parasites seen |  | 286 |  | 7** |  |  |  | 97.6 (94.9-98.9) |  |  |  |  |
| No parasites seen |  | 95 |  | 1 |  |  |  |  |  |  |  |  |
| *P. vivax* |  | 80 |  | 1 |  |  |  |  |  |  |  |  |
| *P. ovale* |  | 80 |  | 1 |  |  |  |  |  |  |  |  |
| *P. malariae* |  | 31 |  | 4 |  |  |  |  |  |  |  |  |
|  |  |  |  |  |  |  |  |  |  |  |  |  |

* As defined in Table 1: reading of a HRP-2 line or both the HRP-2 and pLDH lines

**PCR confirmed the microscopic identification in all seven samples except for a mixed *P. malariae/P. falciparum* infection in a microscopic *P. malariae* sample, adjusted specificity = 98.0% (95.4-99.1)
