# Supplementary material for: Test characteristics of two rapid antigen detection tests (SD FK50 and SD FK60) for the diagnosis of malaria in returned travellers
Source: Malar J. 2009 May 5;8:90. doi: 10.1186/1475-2875-8-90 (PMC2688521; doi:10.1186/1475-2875-8-90)
Supplement: Additional file 3 — Test characteristics of the FK60 for non-falciparum species according to parasite densities. [file 1475-2875-8-90-S3.doc]

**Additional file 3**

**Test characteristics of the FK60 for non-falciparum species according to parasite densities (n = 610, 4 mixed infections not included)**

| **Result by microscopy** |  | Numbers |  | Positive  by FK60* |  | Sensitivity in %  (95% C.I.) |  | Specificity in %  (95% C.I.) |  | Positive  Likelihood Ratio |  | Negative  Likelihood Ratio |
| --- | --- | --- | --- | --- | --- | --- | --- | --- | --- | --- | --- | --- |
|  |  |  |  |  |  |  |  |  |  |  |  |  |
| **Test characteristics for non-falciparum samples** |  |  |  |  |  |  |  |  |  |  |  |  |
| All samples combined |  | 191 |  | 145 |  | 75.9 (69.4-81.5) |  |  |  | ∞ |  | 0.24 |
|  |  |  |  |  |  |  |  |  |  |  |  |  |
| *P. vivax*, all samples |  | 80 |  | 70 |  | 87.5 (78.3-93.3) |  |  |  | ∞ |  | 0.13 |
| Parasite density ≤ 500/µl |  | 26 |  | 20 |  | 76.9 (57.6-89.3) |  |  |  | ∞ |  | 0.23 |
| Parasite density > 500/µl |  | 54 |  | 50 |  | 92.6 (82.0-97.6) |  |  |  | ∞ |  | 0.07 |
|  |  |  |  |  |  |  |  |  |  |  |  |  |
| *P. ovale*, all samples |  | 80 |  | 61 |  | 76.3 (65.8-84.3) |  |  |  | ∞ |  | 0.24 |
| Parasite density ≤ 500/µl |  | 38 |  | 23 |  | 60.5 (44.7-77.4) |  |  |  | ∞ |  | 0.39 |
| Parasite density > 500/µl |  | 42 |  | 38 |  | 90.5 (77.3-96.8) |  |  |  | ∞ |  | 0.10 |
|  |  |  |  |  |  |  |  |  |  |  |  |  |
| *P. malariae*, all samples |  | 31 |  | 14** |  | 45.2 (29.2-62.2) |  |  |  | ∞ |  | 0.55 |
| Parasite density ≤ 500/µl |  | 11 |  | 2 |  | 18.2 (0.04-48.9) |  |  |  | ∞ |  | 0.82 |
| Parasite density > 500/µl |  | 20 |  | 12 |  | 60.0 (38.6-78.2) |  |  |  | ∞ |  | 0.40 |
|  |  |  |  |  |  |  |  |  |  |  |  |  |
| *P. falciparum* and no parasites seen | | 419 |  | 0 |  |  |  | 100 (99.2-100.0) |  |  |  |  |
| No parasites seen |  | 95 |  | 0 |  |  |  |  |  |  |  |  |
| *P. falciparum* |  | 324 |  | 0 |  |  |  |  |  |  |  |  |
|  |  |  |  |  |  |  |  |  |  |  |  |  |

* As defined in Table 2: reading of a unique pLDH line

**PCR confirmed the microscopic identification in discordant samples except for a mixed *P. malariae/P. falciparum* infection in a microscopic *P. malariae* sample, adjusted sensitivity for the latter = 48.4% (32.0-65.2)
